# Cardiovascular Event Rates in Statin-Treated Korean Patients with Cardiovascular Disease: Estimates from a Real-World Population Using Electronic Medical Record Data

**DOI:** 10.1007/s10557-021-07255-2

**Published:** 2021-10-08

**Authors:** Osung Kwon, Wonjun Na, Jaehee Hur, Ju Hyeon Kim, Tae Joon Jun, Hee Jun Kang, Hojoon Lee, Young-Hak Kim

**Affiliations:** 1 Division of Cardiology, Department of Internal Medicine, Eunpyeong St. Mary’s Hospital, College of Medicine, The Catholic University of Korea, Seoul, Republic of Korea; 2 Division of Cardiology, Department of Internal Medicine, Asan Medical Center, University of Ulsan College of Medicine, Seoul, Republic of Korea; 3 Department of Medical Science, Asan Medical Institute of Convergence Science and Technology, Asan Medical Center, University of Ulsan College of Medicine, Seoul, Republic of Korea; 4 Health Innovation Big Data Center, Asan Institute for Life Sciences, Asan Medical Center, Seoul, Republic of Korea; 5 Amgen Korea, Seoul, Republic of Korea

**Keywords:** Cardiovascular disease, Secondary prevention, Dyslipidemia, Cholesterol, PCSK9 inhibitors, Electronic medical records

## Abstract

**Purpose:**

To estimate the risk of recurrent cardiovascular events in a real-world population of very high-risk Korean patients with prior myocardial infarction (MI), ischemic stroke (IS), or symptomatic peripheral artery disease (sPAD), similar to the Further cardiovascular OUtcomes Research with proprotein convertase subtilisin–kexin type 9 Inhibition in subjects with Elevated Risk (FOURIER) trial population.

**Methods:**

This retrospective study used the Asan Medical Center Heart Registry database built on electronic medical records (EMR) from 2000 to 2016. Patients with a history of clinically evident atherosclerotic cardiovascular disease (ASCVD) with multiple risk factors were followed up for 3 years. The primary endpoint was a composite of MI, stroke, hospitalization for unstable angina, coronary revascularization, and all-cause mortality.

**Results:**

Among 15,820 patients, the 3-year cumulative incidence of the composite primary endpoint was 15.3% and the 3-year incidence rate was 5.7 (95% CI 5.5–5.9) per 100 person-years. At individual endpoints, the rates of deaths, MI, and IS were 0.4 (0.3–0.4), 0.9 (0.8–0.9), and 0.8 (0.7–0.9), respectively. The risk of the primary endpoint did not differ significantly between recipients of different intensities of statin therapy. Low-density lipoprotein cholesterol (LDL-C) goals were only achieved in 24.4% of patients during the first year of follow-up.

**Conclusion:**

By analyzing EMR data representing routine practice in Korea, we found that patients with very high-risk ASCVD were at substantial risk of further cardiovascular events in 3 years. Given the observed risk of recurrent events with suboptimal lipid management by statin, additional treatment to control LDL-C might be necessary to reduce the burden of further cardiovascular events for very high-risk ASCVD patients.

**Supplementary Information:**

The online version contains supplementary material available at 10.1007/s10557-021-07255-2.

## Introduction

Cardiovascular disease (CVD) is the leading cause of death globally [1]. Patients who experience a cardiovascular event are at increased risk of further events [2–5]. Dyslipidemia is known to be a major risk factor for CVD [6, 7], and management of low-density lipoprotein cholesterol (LDL-C) levels is critical in patients at high risk of CVD [8, 9]. High LDL-C is not only a risk factor for primary CVD events, but also for secondary or recurrent CVD events in high-risk CVD patients [10–12]. Statins have been the mainstay of LDL-C lowering therapy, but there is still a residual risk of CVD attributable to high LDL-C. Thus, additional drugs may be needed to achieve adequate control in some patients [8].

The Further cardiovascular OUtcomes Research with proprotein convertase subtilisin–kexin type 9 (PCSK9) Inhibition in subjects with Elevated Risk (FOURIER) trial demonstrated the risk of secondary CVD events among high-risk patients and showed that adding evolocumab to statin therapy reduced the risk of recurrent cardiovascular events in patients with clinically evident atherosclerotic cardiovascular disease (ASCVD) [13]. The majority of data about the risk of cardiovascular events in patients with existing CVD and new lipid-lowering treatments come from clinical trials. A few retrospective studies have estimated the risk of cardiovascular events in very high-risk patients who met the inclusion criteria of the FOURIER trial in real-world settings (FOURIER-like populations) in Europe and the USA [14–16]. As such, there is a need to obtain data from routine clinical practice, in order to explore any evidence gaps between the real-world setting and clinical trials, particularly in Asian populations.

Thus, the purpose of the current study, using electronic medical records (EMR) data from a tertiary hospital, was to obtain information about the clinical characteristics and to estimate the rates of cardiovascular outcomes in very high-risk Korean patients with prior myocardial infarction (MI), ischemic stroke (IS), or symptomatic peripheral artery disease (sPAD) who met the inclusion criteria for the FOURIER trial. In addition, we tried to evaluate the real-world treatment pattern of LDL-C for the very high-risk Korean population.

## Methods

### Study Design

A retrospective observational cohort study was conducted using EMR data from a large tertiary hospital in Korea. The study included adult patients at high risk of cardiovascular events and receiving statin therapy, who were followed for the occurrence of subsequent cardiovascular events. The study period was from 1 January 2000 to 30 November 2016.

Data were obtained from the Asan biomedical research environment database, which is a data warehouse system [17] for the Asan Medical Center (AMC), one of the largest medical institutions in the Republic of Korea. The Asan biomedical research environment database includes de-identified information for 4 million patients and is updated every 3 days. The AMC Heart Registry (AMC-HR) was constructed using fields of structured or unstructured EMR data extracted from the Asan biomedical research environment database using structured query language queries. The AMC-HR was standardized and validated to allow EMR-based research [18]. The AMC-HR comprised 571,157 subjects who met the inclusion criteria of inpatient or outpatient encounters in the cardiology, cardiac surgery, or emergency departments for established or suspected heart disease between 1 January 2000 and 30 November 2016. Collection of data based on implied consent was approved by the AMC institutional review board. De-identification of data was performed in line with the health insurance portability and accountability act for Korea.

### Study Population

The study included patients from the AMC-HR who had a history of clinically evident cardiovascular disease (MI, IS, or sPAD) and at least 1 major risk factor or at least 2 minor risk factors (similar to the criteria for the FOURIER trial; Table 1) between 1 January 2000 and 30 November 2013. The index date was defined as the date of the first diagnosis of MI, IS, or sPAD. Definitions and associated ICD-10 or claim codes for the inclusion criteria variables are provided in Online Resource 1. The study population was stratified into three subcohorts according to the index cardiovascular event (MI, IS, and sPAD cohorts) and intensity of statin, to facilitate a detailed analysis.

**Table 1:** Key inclusion criteria for the AMC-HR study and comparison with those for the FOURIER study

FOURIER inclusion criteria [13]	AMC-HR inclusion criteria
Major risk factors (1 required) • Diabetes (type 1 or type 2) • Age ≤ 65 and ≥ 85 years • MI or non-hemorrhagic stroke within 6 months of screening • Additional diagnosis of MI or non-hemorrhagic stroke • Current daily cigarette smoking • History of sPAD	Major risk factors (1 required) • Diabetes (type 1 or type 2) • Age ≤ 65 and ≥ 85 years • MI or IS diagnosis within last 6 months • History of > 1 MI or IS event • Current smoking • History of sPAD
Minor risk factors (2 required) • History of non-MI-related coronary revascularization • Residual coronary artery disease • HDL-C < 40 mg/dL for men or < 50 mg/dL for women • High-sensitivity CRP > 2.0 mg/L • Metabolic syndrome, defined as the presence of at least 3 of the following 5 conditions: - Elevated waist circumference - Triglycerides ≥ 150 mg/dL - Low HDL-C (as defined above) - Fasting glucose ≥ 100 mg/dL - Hypertension	Minor risk factors (2 required) • History of non-MI-related coronary revascularization • HDL-C < 40 mg/dL for men or < 50 mg/dL for women • High-sensitivity CRP > 2.0 mg/L • Metabolic syndrome, defined as the presence of at least 2 of the following 4 conditions: - BMI ≥ 30^a^ - Triglycerides ≥ 150 mg/dL - Low HDL-C (as defined above) - Hypertension

*AMC-HR* Asan Medical Center Heart Registry, *BMI* body mass index, *CAD* coronary artery disease, *CPRD* Clinical Practice Research Datalink, *CRP* C-reactive protein, *FOURIER* Further cardiovascular OUtcomes Research with PCSK9 Inhibition in subjects with Elevated Risk, *HDL-C* high-density lipoprotein cholesterol, *IS* ischemic stroke, *MI* myocardial infarction, *SI* International System of Units, *sPAD* symptomatic peripheral artery disease

^a^Modified criteria for central obesity were based on the new International Diabetes Federation definition

Patients were excluded if they were aged < 40 or > 85 years, had a recent MI or stroke within 4 weeks of the index date, did not have blood LDL-C levels available, were not receiving a statin, had uncontrolled hypertension, or had a history of hemorrhagic stroke at any time.

### Outcomes

The primary endpoint was a composite of MI, stroke, hospitalization for unstable angina (UA), coronary revascularization (coronary artery bypass grafting [CABG] or percutaneous coronary intervention [PCI]), and all-cause mortality at 3-year follow-up. This mirrored the primary endpoint in the FOURIER trial except that cardiovascular-related death was replaced with all-cause mortality for the current study. The secondary endpoint was a composite of MI, stroke, and all-cause mortality.

The follow-up period continued through to 30 November 2016, to ensure that all patients had the opportunity for a 3-year follow-up evaluation. Mortality was captured through documentation of mortality in EMR based upon national health insurance information. Cardiovascular events, including MI, UA, and stroke, were captured by identifying hospitalizations with a relevant primary or secondary ICD-10 diagnosis and adjudicating the event in conjunction with related laboratory results and reports on imaging studies by cardiologists and neurologists. Repeat revascularization was identified by the presence of cost codes for CABG or PCI, with final confirmation by a cardiologist based on the notes for the procedure. Definitions and associated ICD-10 or claim codes for the individual outcome variables are provided in Online Resource 1.

### Statistical Analysis

Continuous variables were presented as mean values with standard deviation and categorical variables as numbers with percentages. Continuous variables were compared using the Student *t* test or the Wilcoxon rank-sum test, and categorical variables were compared using *χ*^2^ statistics or Fisher’s exact test, as appropriate.

Incidence rates for the outcomes of interest were calculated as the number of first events divided by the total follow-up person-time at risk until the event or the end of 3-year follow-up, expressed as rates per 100 person-years. Cumulative incidence and survival curves were generated using the Kaplan–Meier method.

Multivariable Cox-proportional hazard regression models were applied to calculate hazard ratios (HRs) and 95% confidence intervals (CIs) for the primary and secondary outcomes and for all-cause mortality, according to statin dose. The models included adjustment for age, gender, renal function (estimated glomerular filtration rate [eGFR] < 60 mL/min/1.73 m^2^), hypertension, diabetes, current smoking, and medications (including non-statin lipid-lowing agents, aspirin or P2Y12 inhibitors, beta-blockers, and RAAS inhibitors). Cox-proportional hazard regression models were also used to identify risk factors contributing to the primary and secondary outcomes and to all-cause mortality.

A two-tailed *p* value < 0.05 was considered statistically significant. Statistical analyses were performed using R software version 3.2.2 (R Foundation for Statistical Computing, Vienna, Austria; www.r-project.org).

## Results

**Fig. 1:**
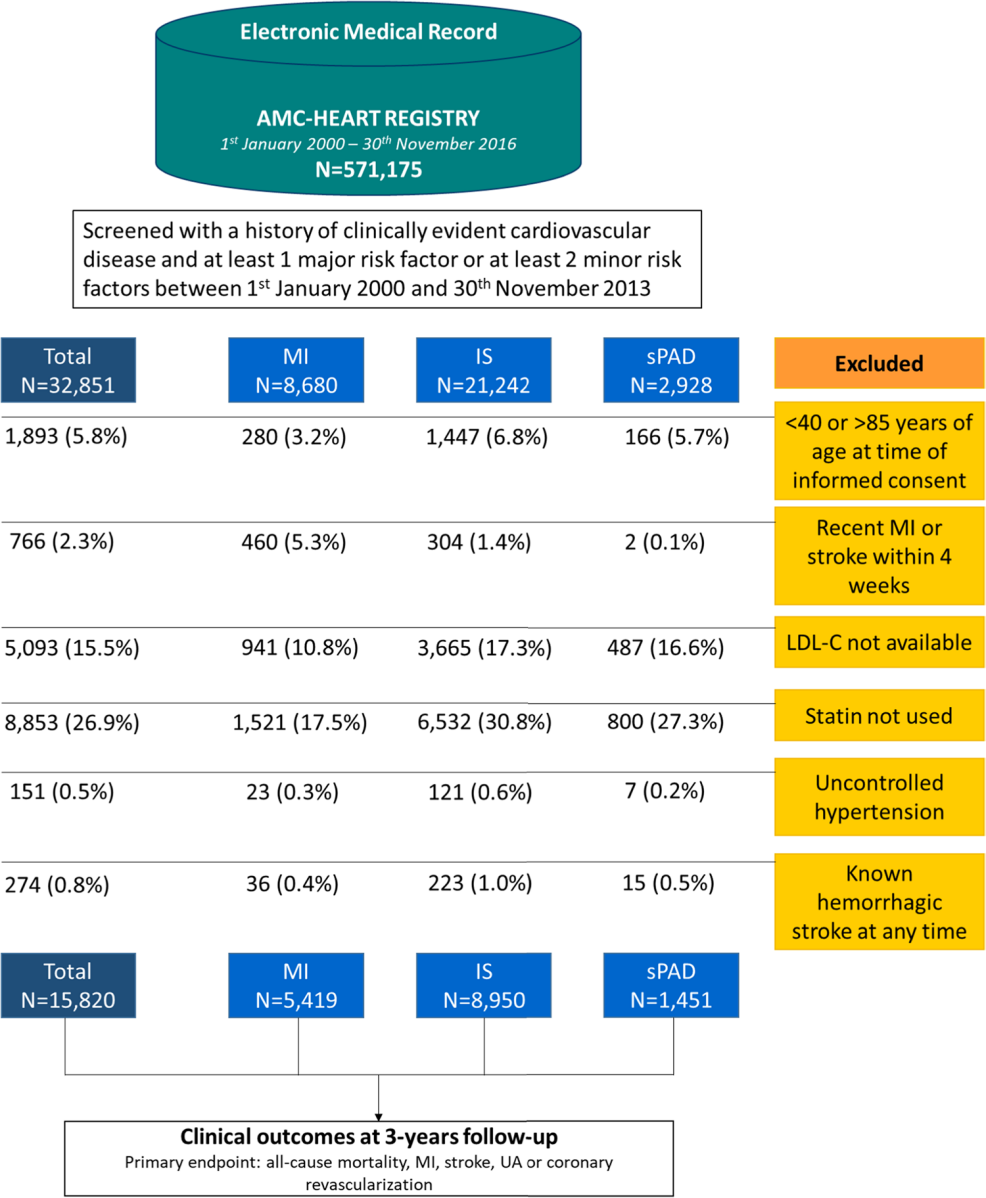
Flow of study participants. AMC, Asan Medical Center; IS, ischemic stroke; LDL-C, low-density lipoprotein cholesterol; MI, myocardial infarction; sPAD, symptomatic peripheral artery disease

Among subjects enrolled in the AMC-HR between 1 January 2000 and 30 November 2013, a total of 32,850 had a history of clinically evident ASCVD and at least 1 major risk factor or at least 2 minor risk factors. After screening, 17,030 of these patients were excluded because they were aged < 40 or > 85 years, had a recent MI or stroke, did not have blood LDL-C levels available, were not receiving a statin, had uncontrolled hypertension, or had a history of hemorrhagic stroke. This resulted in a final study cohort of 15,820 patients, comprising 5419 with a history of MI, 8950 with a history of IS, and 1451 with sPAD (Fig. 1).

The baseline characteristics of the population are summarized in Table 2. In general, the characteristics of the AMC-HR cohort were consistent with those of the FOURIER population, with a few exceptions. A history of MI was less common in AMC-HR than in FOURIER (34.3% versus 81.3% in the FOURIER placebo group), whereas a history of IS was more common (56.6% versus 19.2%). Most patients in AMC-HR were receiving moderate-intensity statin therapy at the time of the index event (81.8%), whereas in FOURIER most were on high-intensity statins (69.1%). Mean LDL-C level was slightly higher in the AMC-HR cohort than in the FOURIER population (104 versus 92 mg/dL), whereas triglyceride and lipoprotein(a) levels tended to be lower in the AMC-HR population. Mean body weight was lower in the AMC-HR cohort than in FOURIER (64.9 ± 14.6 kg versus 85.5 ± 17.4 kg in the FOURIER placebo group).

**Table 2:** Baseline characteristics for the FOURIER-like AMC-HR study population and for the FOURIER study population

	AMC-HR study (FOURIER-like population)	FOURIER study [13]	
	Total (*n* = 15,820)	Evolocumab (*n* = 13,784)	Placebo (*n* = 13,780)
Age, years, mean ± SD	63.2 ± 10.2	62.5 ± 9.1	62.5 ± 8.9
Men	10,665 (67.4)	10,397 (75.4)	10,398 (75.5)
Weight, kg, mean ± SD	64.9 ± 14.6	85.0 ± 17.3	85.5 ± 17.4
BMI, kg/m^2^, mean ± SD	24.7 ± 8.4	–	–
SBP, mmHg, mean ± SD	129.7 ± 21.5	–	–
DBP, mmHg, mean ± SD	74.8 ± 12.5		
eGFR < 60 mL/min/1.73 m^2^	2773 (17.6)	–	–
*Lipids, median (IQR)*			
LDL-C, mg/dL	104 (78–132)	92 (80–109)	92 (80–109)
Total cholesterol, mg/dL	164 (134–197)	168 (151–188)	168 (151–189)
HDL-C, mg/dL	42 (35–51)	44 (37–53)	44 (37–53)
Triglycerides, mg/dL	118 (85–166)	134 (101–183)	133 (99–181)
Lipoprotein(a), nmol/L	23.3 (12.1–42.5)	37 (13–166)	37 (13–164)
*Cardiovascular risk factors*			
Hypertension	11,881 (75.1)	11,045 (80.1)	11,039 (80.1)
Diabetes	6651 (42.0)	5054 (36.7)	5027 (36.5)
Current smoking	3439 (21.7)	3854 (28.0)	3923 (28.5)
*Cardiovascular disease*			
MI	5419 (34.3)	11,145 (80.9)	11,206 (81.3)
IS	8950 (56.6)	2686 (19.5)	2651(19.2)
sPAD	1451 (9.2)	1858 (13.5)	1784 (12.9)
*Statin use:* s*tatin intensity at the index event*			
Low intensity	1919 (12.1)	38 (0.3)	31 (0.2)
Moderate intensity	12,936 (81.8)	4161 (30.2)	4231 (30.7)
High intensity	965 (6.1)	9585 (69.5)	9518 (69.1)
*Other medication use*			
Non-statin lipid-lowering treatment			
Ezetimibe	1919 (12.1)	726 (5.3)	714 (5.2)
Fibrate	927 (5.9)	–	–
Niacin	0 (0.0)	–	–
Cholestyramine	17 (0.1)	–	–
Aspirin or P2Y12 inhibitors	14,904 (94.2)	12,766 (92.7)	12,666 (92.0)
Beta-blockers	10,340 (65.4)	10,441 (75.8)	10,374 (75.4)
RAAS inhibitors (ACE inhibitors or ARBs, aldosterone antagonists, or both)	11,420 (72.2)	10,803 (78.4)	10,730 (77.9)

Data are presented as *n* (%) unless otherwise indicated

*ACE* angiotensin-converting enzyme, *ACR* albumin-to-creatinine ratio, *AMC* Asan Medical Center Heart Registry, *ARB* angiotensin-receptor blocker, *BMI* body mass index, *BP* blood pressure, *CABG* coronary artery bypass graft, *eGFR* estimated glomerular filtration rate, *HDL-C* high-density lipoprotein cholesterol, *hsCRP* high-sensitivity C-reactive protein, *IQR* interquartile range, *IS* ischemic stroke, *LDL-C* low-density lipoprotein cholesterol, *MI* myocardial infarction, *PCI* percutaneous coronary intervention, *RAAS* renin–angiotensin–aldosterone system, *SBP* systolic blood pressure, *SD* standard deviation, *sPAD* symptomatic peripheral artery disease

The baseline characteristics for subcohorts of the AMC-HR population, based on index cardiovascular event or on intensity of statin treatment are summarized in Online Resource 2. Patients in the subcohort with a history of MI were less likely to have hypertension than the subcohorts with a history of IS or sPAD (68.3% versus 78.7% and 78.3%) and more likely to be receiving beta-blockers (90.5% versus 49.7% and 68.3%) and RAAS inhibitors (79.4% versus 67.7% and 72.7%). The IS subcohort had a numerically higher proportion of women (39.4% versus 24.0% and 22.5%), a higher median LDL-C level (109 versus 99.4 and 95.4 mg/dL), and a smaller proportion on ezetimibe (9.1% versus 15.5% and 18.0%) than the MI and sPAD subcohorts. The sPAD subcohort had a greater proportion of patients with diabetes (55.8% versus 39.3% and 41.5%) and patients receiving low-intensity statins (20.1% versus 10.9% and 11.6%) compared with the MI and IS subcohorts. The subcohorts who were receiving low-/moderate-intensity statin therapy had a higher median LDL-C level (105 versus 99.8 mg/dL), lower triglyceride level (117 versus 123 mg/dL), greater proportion of patients with a history of IS (59.1% versus 45.1%), and smaller proportion on beta-blocker treatment (63.3% versus 74.7%), compared with the subcohorts who were receiving high-intensity statins or statins plus ezetimibe.

### Cardiovascular Events

**Fig. 2:**
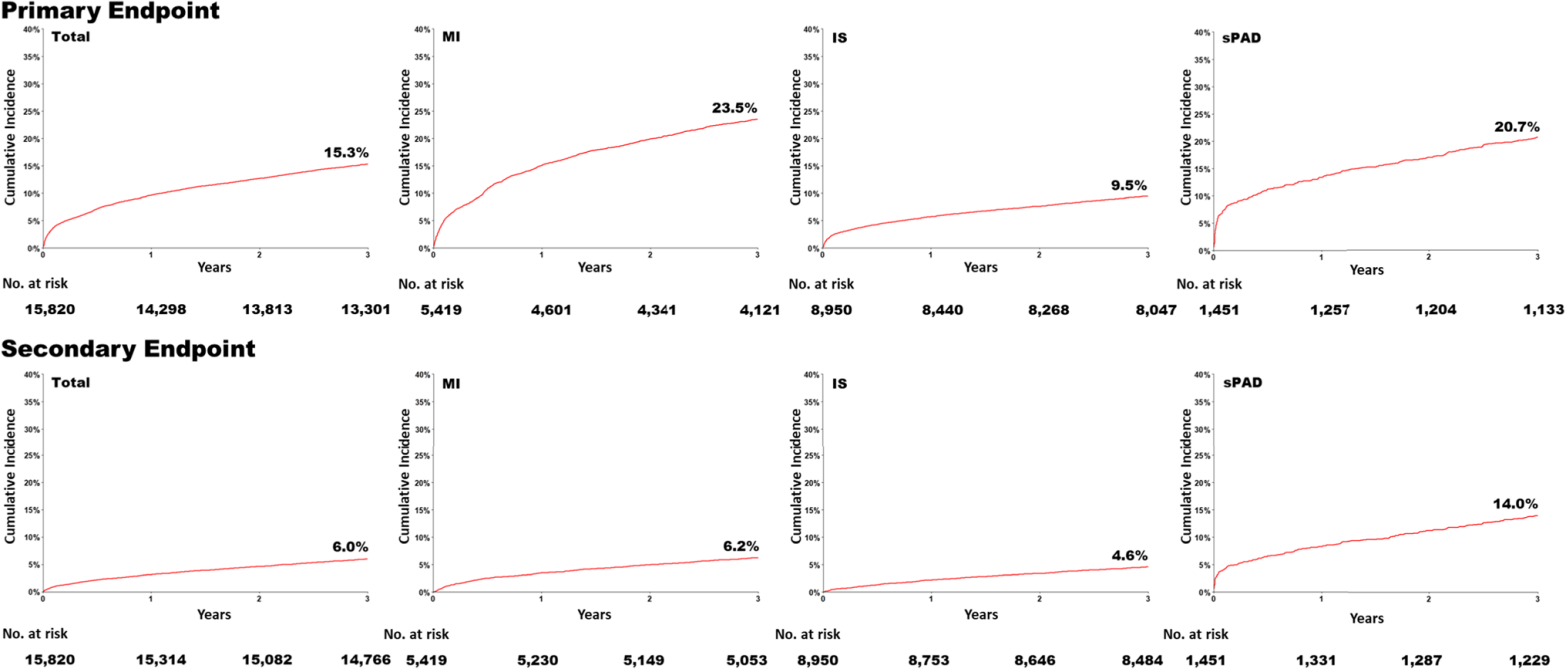
Three-year cumulative incidences of the primary and secondary endpoints in AMC-HR (for the total cohort and for the subcohorts with a history of MI, IS, or sPAD). Primary endpoint: composite of MI, stroke, hospitalization for UA, revascularization, and all-cause mortality. Secondary endpoint: composite of MI, stroke, and all-cause mortality. IS, ischemic stroke; MI, myocardial infarction; sPAD, symptomatic peripheral artery disease; UA, unstable angina

The cumulative incidences of the primary and secondary endpoints are summarized in Fig. 2, for the overall AMC-HR cohort and for the subcohorts with a history of MI, IS, or sPAD. For the total cohort, the 3-year cumulative incidence of the composite primary endpoint (MI, stroke, hospitalization for UA, coronary revascularization, and all-cause mortality) was 15.3%, and the 3-year cumulative incidence of the secondary composite endpoint (MI, stroke, and all-cause mortality) was 6.0%. The 3-year cumulative incidences of the composite primary and secondary endpoints were 23.5% and 6.2% in the MI subcohort, 9.5% and 4.6% in the IS subcohort, and 20.7% and 14.0% in the sPAD subcohort.

Cardiovascular event rates are presented in Table 3. For the overall AMC-HR cohort, the 3-year incidence rate for the primary composite endpoint was 5.7 (95% CI 5.5–5.9) per 100 person-years and for the secondary composite endpoint was 2.1 (95% CI 2.0–2.2) per 100 person-years. Among the individual components of the primary endpoint, 3-year incidence rates in the overall cohort ranged from 0.4 (95% CI 0.3–0.4) for MI to 2.8 (95% CI 2.6–2.9) for revascularization procedures. After stratification by subcohort, revascularization and hospitalization for UA was most frequent in the MI cohort, while stroke and all-cause mortality were the second most frequent events in the sPAD and IS cohorts, respectively.

**Table 3:** Three-year cardiovascular event rates in AMC-HR (for the total cohort and for the subcohorts with a history of MI, IS, or sPAD)

Endpoint	Total (*n* = 15,820)			MI cohort (*n* = 5419)			IS cohort (*n* = 8950)			sPAD cohort (*n* = 1451)		
	Events	P-Y	Rate per 100 P-Y (95% CI)	Events	P-Y	Rate per 100 P-Y (95% CI)	Events	P-Y	Rate per 100 P-Y (95% CI)	Events	P-Y	Rate per 100 P-Y (95% CI)
Primary endpoint^a^	2421	42,401	5.7 (5.5–5.9)	1274	13,576	9.4 (8.9–9.9)	847	25,123	3.4 (3.1–3.6)	300	3703	8.1 (7.2–9.0)
Secondary endpoint^b^	951	45,670	2.1 (2.0–2.2)	339	15,605	2.2 (1.9–2.4)	409	26,128	1.6 (1.4–1.7)	203	3937	5.2 (4.5–5.9)
*Individual endpoints*												
All-cause mortality	403	46,728	0.9 (0.8–0.9)	155	15,949	1.0 (0.8–1.1)	176	26,541	0.7 (0.6–0.8)	72	4238	1.7 (1.3–2.1)
MI	176	47,135	0.4 (0.3–0.4)	25	16,211	0.2 (0.1–0.2)	106	26,649	0.4 (0.3–0.5)	45	7275	0.6 (0.4–0.8)
Stroke	445	46,625	1.0 (0.9–1.0)	180	15,925	1.1 (1.0–1.3)	157	26,597	0.6 (0.5–0.7)	108	4102	2.6 (2.1–3.1)
Ischemic	372	46,759	0.8 (0.7–0.9)	154	15,973	1.0 (0.8–1.1)	126	26,648	0.5 (0.4–0.6)	92	4138	2.2 (1.8–2.7)
Hemorrhagic	73	47,321	0.2 (0.1–0.2)	26	16,206	0.2 (0.1–0.2)	31	26,803	0.1 (0.1–0.2)	16	4313	0.4 (0.2–0.6)
Hospitalization for UA	708	46,156	1.5 (1.4–1.6)	491	15,319	3.2 (2.9–3.5)	159	26,579	0.6 (0.5–0.7)	58	4258	1.4 (1.0–1.7)
Revascularization (CABG/PCI)	1234	44,709	2.8 (2.6–2.9)	689	14,753	4.7 (4.3–5.0)	433	25,866	1.7 (1.5–1.8)	112	4090	2.7 (2.2–3.2)

*CABG* coronary artery bypass graft, *CI* confidence interval, *IS* ischemic stroke, *MI* myocardial infarction, *PCI* percutaneous coronary intervention, *P-Y* person-years, *sPAD* symptomatic peripheral artery disease, *UA* unstable angina

^a^Primary endpoint: composite of MI, stroke, hospitalization for UA, revascularization, and all-cause mortality

^b^Secondary endpoint: composite of MI, stroke, and all-cause mortality

The risk of meeting the primary or secondary endpoints during the 3 years of observation did not differ between recipients of different intensities of statin therapy (Table 4). Nonetheless, there was a trend towards a reduced risk of all-cause mortality in high-intensity treatment, as indicated by hazard ratios of 0.66 (95% CI 0.38–1.13) for high- versus low-intensity statin use and 0.73 (95% CI 0.50–1.07) for high-intensity statin or any statin plus ezetimibe versus low-/moderate-intensity statin without ezetimibe. No association between the baseline LDL-C level and recurrent cardiovascular events was observed (Table 4).

**Table 4:** Risk of meeting the primary or secondary endpoints or all-cause mortality, according to the intensity of statin therapy and baseline LDL-C level

	Primary endpoint^a^				Secondary endpoint^b^				All-cause mortality			
	Event	P-Y	HR (95% CI)	*p*	Event	P-Y	HR (95% CI)	*p*	Event	P-Y	HR (95% CI)	*p*
*Statin use*^c^												
Low intensity	314	5135	1 (reference)		121	5547	1 (reference)		49	5681	1 (reference)	
Moderate intensity	1975	34,661	1.00 (0.88–1.13)	0.972	763	37,368	0.91 (0.75–1.11)	0.356	335	38,190	0.88 (0.64–1.20)	0.413
High intensity	132	2606	0.86 (0.70–1.06)	0.152	66	2754	1.04 (0.76–1.41)	0.805	19	2857	0.66 (0.38–1.13)	0.126
*Statin use*^d^												
Low or moderate intensity (without ezetimibe)	1935	35,030	1 (reference)		782	37,595	1 (reference)		357	38,401	1 (reference)	
High intensity or any statin with ezetimibe	486	7371	0.93 (0.81–1.06)	0.276	169	8074	1.05 (0.85–1.31)	0.633	46	8327	0.73 (0.50–1.07)	0.108
Baseline LDL-C (mg/dL)												
Q1 ≤ 78	660	10,678	1 (reference)		287	11,496	1 (reference)		126	11,816	1 (reference)	
78 < Q2 ≤ 104	594	10,448	1.01 (0.86–1.19)	0.891	244	11,228	1.09 (0.84–1.40)	0.521	98	11,506	0.88 (0.60–1.27)	0.481
104 < Q3 ≤ 132	618	10,667	1.12 (0.95–1.32)	0.166	199	11,610	0.95 (0.73–1.23)	0.699	82	11,825	0.80 (0.55–1.17)	0.254
Q4 > 132	549	10,609	1.08 (0.92–1.27)	0.369	221	11,336	1.11 (0.86–1.44)	0.417	97	11,581	0.98 (0.67–1.43)	0.919

*CI* confidence interval, *eGFR* estimated glomerular filtration rate, *HR* hazard ratio, *MI* myocardial infarction, *LDL-C* low-density lipoprotein cholesterol, *P-Y* person-years, *RAAS* renin–angiotensin–aldosterone system, *UA* unstable angina

^a^Primary endpoint: composite of MI, stroke, hospitalization for UA, revascularization, and all-cause mortality

^b^Secondary endpoint: composite of MI, stroke, and all-cause mortality

^c^Statin use, 3 groups (low, moderate, high) adjusted for age, gender, LDL-C, diabetes, smoking, any non-statin lipid-lowering treatment, aspirin or P2Y12 inhibitors, beta-blockers, RAAS inhibitors, eGFR, and statin use (3 groups)

^d^Statin use, 2 groups (low or moderate vs. high or any statin with ezetimibe) adjusted for age, gender, LDL-C, diabetes, smoking, any non-statin lipid-lowering treatment, aspirin or P2Y12 inhibitors, beta-blockers, RAAS inhibitors, eGFR, and statin use (2 groups)

### Risk Factors

Results of the analysis of potential risk factors contributing to cardiovascular outcomes within 3 years in the AMC-HR cohort are summarized in Table 5. Factors significantly associated with an increased risk of meeting the primary endpoint included male sex, diabetes, reduced renal function (eGFR < 60 mL/min/1.73 m^2^), and receiving non-statin lipid-lowering treatment, aspirin or P2Y12 inhibitors, beta-blockers, or RAAS inhibitors. Factors associated with an increased risk of meeting the secondary endpoint included age ≥ 65 years, diabetes, eGFR < 60 mL/min/1.73 m^2^, and treatment with beta-blockers or RAAS inhibitors. Factors significantly associated with an increased risk of all-cause mortality included age ≥ 65 years, diabetes, eGFR < 60 mL/min/1.73 m^2^, and treatment with beta-blockers.

**Table 5:** Contribution of risk factors to cardiovascular outcomes in AMC-HR

	Primary endpoint				Secondary endpoint				All-cause mortality			
	Events	P-Y	HR (95% CI)	*p*	Events	P-Y	HR (95% CI)	*p*	Events	P-Y	HR (95% CI)	*p*
*Demographics*												
Age												
< 65 years	1241	22,394	1 (reference)		348	24,328	1 (reference)		122	24,747	1 (reference)	
≥ 65 years	1180	20,007	**1.06 (0.98–1.16)**	0.147	603	21,342	**1.74 (1.52–2.00)**	< 0.001	281	21,981	**2.15 (1.72–2.68)**	< 0.001
Sex												
Women	657	14,081	1 (reference)		310	14,867	1 (reference)		139	15,196	1 (reference)	
Men	1764	28,320	**1.25 (1.14–1.37)**	< 0.001	641	30,802	**1.11 (0.96–1.28)**	0.165	264	31,532	**1.10 (0.88–1.36)**	0.400
*Cardiovascular risk factors*												
Hypertension												
No	462	10,804	1 (reference)		148	11,512	1 (reference)		78	11,656	1 (reference)	
Yes	1959	31,597	**1.01 (0.90–1.12)**	0.900	803	34,158	**1.14 (0.95–1.38)**	0.154	325	35,072	**0.80 (0.61–1.05)**	0.105
Diabetes												
No	1174	25,044	1 (reference)		410	26,714	1 (reference)		162	27,194	1 (reference)	
Yes	1247	17,357	**1.24 (1.14–1.35)**	< 0.001	541	18,955	**1.45 (1.27–1.66)**	< 0.001	241	19,533	**1.66 (1.35–2.04)**	< 0.001
Current smoking												
No	1879	33,193	1 (reference)		760	35,705	1 (reference)		330	36,534	1 (reference)	
Yes	542	9208	**1.01 (0.91–1.11)**	0.871	191	9965	**1.06 (0.90–1.25)**	0.494	73	10,194	**1.04 (0.79–1.35)**	0.788
eGFR (mL/min/1.73 m^2^)												
≥ 60	1813	35,271	1 (reference)		606	37,932	1 (reference)		218	38,678	1 (reference)	
< 60	608	7130	**1.22 (1.11–1.34)**	< 0.001	345	7737	**1.97 (1.71–2.26)**	< 0.001	185	8050	**3.03 (2.46–3.74)**	< 0.001
*Other medication use*												
Any non-statin lipid-lowering treatment												
No	1904	35,458	1 (reference)		790	37,948	1 (reference)		357	38,775	1 (reference)	
Yes	517	6943	**1.13 (1.02–1.25)**	0.023	161	7721	**0.86 (0.72–1.03)**	0.097	46	7953	**0.55 (0.40–0.75)**	< 0.001
Aspirin or P2Y12 inhibitors												
No	42	2673	1 (reference)		36	2681	1 (reference)		22	2707	1 (reference)	
Yes	2379	39,789	**2.71 (2.00–3.69)**	< 0.001	915	42,988	**1.20 (0.86–1.68)**	0.289	381	44,021	**0.84 (0.55–1.30)**	0.438
Beta-blockers												
No	311	15,800	1 (reference)		190	16,074	1 (reference)		95	16,252	1 (reference)	
Yes	2110	26,601	**3.17 (2.81–3.58)**	< 0.001	761	29,596	**1.73 (1.47–2.04)**	< 0.001	308	30,475	**1.41 (1.11–1.79)**	0.005
RAAS inhibitors												
No	335	12,552	1 (reference)		139	12,934	1 (reference)		69	13,070	1 (reference)	
Yes	2086	29,849	**1.82 (1.61–2.06)**	< 0.001	812	32,736	**1.55 (1.28–1.87)**	< 0.001	334	33,658	**1.30 (0.98–1.73)**	0.066

*AMC-HR* Asan Medical Center Heart Registry, *CI* confidence interval, *eGFR* estimated glomerular filtration rate, *HR* hazard ratio, *LDL-C* low-density lipoprotein cholesterol, *P-Y* person-years, *RAAS* renin–angiotensin–aldosterone system

### LDL-C Follow-Up

LDL-C levels were re-evaluated within 1 year of the index event in 60.8% of the AMC-HR cohort, with no marked difference seen between subgroups based on the intensity of statin treatment that was being received at the time of the index event (Table 6). LDL-C goals were only achieved in 24.4% of patients within a year; there was a trend towards a higher rate of goal achievement as statin intensity increased (from 18.7% in the low-intensity group to 34.1% in the high-intensity group). Lipid-lowering treatment was intensified in only 0.7% of patients during 1 year of follow-up.

**Table 6:** LDL-C follow-up and alterations in lipid-lowering treatment, according to statin intensity at time of the index event

	Total population	Low-intensity	Moderate-intensity	High-intensity
	*n* = 15,820	*n* = 1919	*n* = 12,936	*n* = 965
LDL-C follow-up between 2 weeks and 1 year	9613 (60.8%)	1124 (58.6%)	7911 (61.2%)	578 (59.9%)
LDL-C < 70 mg/dL or 50% reduction	3867 (24.4%)	359 (18.7%)	3179 (24.6%)	329 (34.1%)
Statin dose increased or ezetimibe added	103 (0.7%)	29 (1.5%)	73 (0.6%)	1 (0.1%)

Data are presented as *n* (%)

*LDL-C* low-density lipoprotein cholesterol

## Discussion

In the current study using EMR data, we aimed to estimate the risk of cardiovascular outcomes among very high-risk Korean patients. The main finding was that the risk of further recurrent cardiovascular events was considerable in very high-risk Korean ASCVD patients managed in a routine practice setting as a 3-year incidence rate for the primary composite endpoint of 5.7 per 100 person-years (cumulative incidence of 15.3%). No significant relationship was found between statin intensity and the risk of meeting the study’s primary or secondary endpoints, where the majority used a moderate-intensity statin. In addition, suboptimal LDL-C management during the follow-up period was observed even on statin treatment.

The 3-year cumulative incidence of 15.3% for this endpoint was consistent with that seen in the placebo group of the FOURIER clinical trial (14.6%) [13], although the primary endpoints in these studies differed slightly, in that the AMC-HR study included all-cause mortality, whereas FOURIER included only cardiovascular death. A few other retrospective studies have also assessed cardiovascular events in a FOURIER-like population in a real-world setting. In a US study, the cumulative incidence of the primary composite endpoint (MI, stroke, coronary revascularization, and cardiovascular death [but not hospitalization for UA]) after 2.2 years was 12.2%, and the cardiovascular event rate was approximately 6.0 per 100 person-years [14]. A Swedish study found that 43.8% of patients experienced one or more new events (using the same composite primary endpoint as FOURIER) during a mean follow-up of 7.3 years, with an incidence rate of 7.0 per 100 person-years [15]. A study in the UK found that 33% of patients experienced a new cardiovascular event (same composite primary endpoint as FOURIER) during a mean follow-up of 5.4 years, with an incidence rate of 7.5 per 100 person-years [16]. With previous literatures, the present study adds to the body of evidence from a real-world setting on the high risk of further cardiovascular events in patients with clinically evident ASCVD and the requirement for additional therapeutic options.

There were a few noteworthy differences between the present cohort driven from AMC-HR and the FOURIER trial population. Firstly, there was a higher prevalence of IS and a lower prevalence of MI in the present cohort compared with FOURIER. It is well-known that Asians have a higher prevalence/incidence of IS than MI [17, 18]. Of note, it can be assumed that if the prevalence of MI in the present cohort had been similar to that in FOURIER, the rate of recurrent events of all population would have been higher than was actually observed because recurrent events occurred more frequently in the MI subgroup compared with other subgroups in AMC-HR cohorts. Secondly, most patients (81.8%) in the present study were receiving moderate-intensity statin therapy at the time of the index event, whereas in FOURIER, approximately 70% of patients were receiving high-intensity statin treatment. In fact, the high prevalence of moderate-intensity statin treatment resulting in suboptimal LDL cholesterol reduction might importantly contribute to the lack of association between statin intensity and clinical outcomes in AMC-HR patients. However, interpretation should be cautious since the majority of patients received moderate statin, leading to statistical limitations for pair comparison. On the other hand, East Asian patients achieve a response to statins at a lower dose than required for Caucasians. Consequently, moderate-intensity statin therapy may be sufficient in most East Asian patients, with high-intensity statin therapy required less often [19, 20]. Hence, no significant relationship between statin intensity and the risk of endpoints in the present study may partly be explained by the ethnic disparity.

Management of LDL-C levels is important for the prevention of ASCVD; however, underutilization of lipid-lowering treatment and failure to intensify treatment is common, even for patients at high cardiovascular risk [21–26]. Measurement of LDL-C levels is often not performed in patients hospitalized with IS, even among those at high risk of future events [25, 27, 28]. Few data are available about the frequency with which lipid levels are measured during follow-up of very high-risk patients receiving lipid-lowering treatment in real-world practice. In the present study based upon EMR data, during the initial screen process, 26.9% of patients were excluded because they were non-statin users (Fig. 1). In addition, during the follow-up period, LDL-C levels were re-evaluated within 1 year in only 60.8% of patients and only 24.4% of patients achieved LDL-C goals within that time. Despite this, < 1% of patients received intensification of statin therapy. These findings suggest inadequate follow-up of LDL-C levels and highlight the need to provide better lipid monitoring. In addition, the substantially low achievement rate of LDL reduction, similarly reported in the previous investigations, indicates that new therapies such as PCSK9 inhibitors may provide an opportunity to reduce the burden of cardiovascular events among the very high ASCVD population [29, 30].

Several limitations should be noted. The first of these was inherent to the retrospective nature and observational design of the analyses. Confirmation of the results in a prospective study would be helpful. Second, although EMR data enabled reliable real-world estimates, findings from a single center cannot be generalized. Third, on analysis of risk factors for primary and secondary endpoints, cardiovascular protective medications such as aspirin, P2Y12 inhibitors, beta-blockers, or RAAS inhibitors were associated with recurrent events. These results may be due to inherent biases of retrospective analysis, but similar findings were also observed in other retrospective studies [14]. It is reasonable to assume that higher risk patients with comorbidities prone to further cardiovascular events were treated more aggressively with various kinds of cardioprotective medications. Additional methodologically rigorous studies are needed to investigate the risk factors on cardiovascular outcomes in a real-world setting. Finally, the event rate was potentially underestimated because clinical events were captured from a single-center EMR database. Linking it with national claims data or health insurance data might possibly capture the events more accurately.

## Conclusion

This study in a routine practice setting in Korea found that very high-risk patients with clinically evident ASCVD were at substantial risk of further cardiovascular events within the subsequent 3 years. In addition, suboptimal LDL-C reduction, even on statin treatment, was observed. These findings may highlight the unmet need and clinical burden among very high-risk ASCVD patients treated with statin therapy and indicate the requirement for additional therapeutic options such as PCSK9 inhibitors.

## Supplementary Information

Below is the link to the electronic supplementary material.

Supplementary file1 (DOCX 27 KB)

## Data Availability

Not applicable.
