# Correction to: Cardiovascular Event Rates in Statin‑Treated Korean Patients with Cardiovascular Disease: Estimates from a Real‑World Population Using Electronic Medical Record Data

**DOI:** 10.1007/s10557-021-07292-x

**Published:** 2021-11-18

**Authors:** Osung Kwon, Wonjun Na, Jaehee Hur, Ju Hyeon Kim, Tae Joon Jun, Hee Jun Kang, Hojoon Lee, Young-Hak Kim

**Affiliations:** 1 Division of Cardiology, Department of Internal Medicine, Eunpyeong St. Mary’s Hospital, College of Medicine, The Catholic University of Korea, Seoul, Republic of Korea; 2 Division of Cardiology, Department of Internal Medicine, Asan Medical Center, University of Ulsan College of Medicine, Seoul, Republic of Korea; 3 Department of Medical Science, Asan Medical Institute of Convergence Science and Technology, Asan Medical Center, University of Ulsan College of Medicine, Seoul, Republic of Korea; 4 Health Innovation Big Data Center, Asan Institute for Life Sciences, Asan Medical Center, Seoul, Republic of Korea; 5 Amgen Korea, Seoul, Republic of Korea

**Correction to: Cardiovascular Drugs and Therapy (2021)**

10.1007/s10557-021-07255-2

The article “Cardiovascular Event Rates in Statin-Treated Korean Patients with Cardiovascular Disease: Estimates from a Real-World Population Using Electronic Medical Record Data” written by Osung Kwon, Wonjun Na^,^ Jaehee Hur^,^ Ju Hyeon Kim^,^ Tae Joon Jun^,^ Hee Jun Kang^,^ Hojoon Lee and Young-Hak Kim was originally published Online First without Open Access. After publication, the author decided to opt for Open Choice and to make the article an Open Access publication. Therefore, the copyright of the article has been changed to © The Author(s) 2021 and the article is forthwith distributed under the terms of the Creative Commons Attribution 4.0 International License (http://creativecommons.org/licenses/by/4.0/), which permits use, duplication, adaptation, distribution and reproduction in any medium or format, as long as you give appropriate credit to the original author(s) and the source, provide a link to the Creative Commons license, and indicate if changes were made.

The original article has been corrected.

Open Access This article is distributed under the terms of the Creative Commons Attribution 4.0 International License (http://creativecommons.org/licenses/by/4.0/), which permits unrestricted use, distribution, and reproduction in any medium, provided you give appropriate credit to the original author(s) and the source, provide a link to the Creative Commons license, and indicate if changes were made. The images or other third party material in this article are included in the article’s Creative Commons licence, unless indicated otherwise in a credit line to the material. If material is not included in the article’s Creative Commons licence and your intended use is not permitted by statutory regulation or exceeds the permitted use, you will need to obtain permission directly from the copyright holder. To view a copy of this licence, visit http:// creativecommons.org/licenses/by/4.0/.

