# Supplementary material for: Cardiovascular Event Rates in Statin-Treated Korean Patients with Cardiovascular Disease: Estimates from a Real-World Population Using Electronic Medical Record Data
Source: Cardiovasc Drugs Ther. 2021 Oct 8;37(1):129–40. doi: 10.1007/s10557-021-07255-2 (PMC9834152; doi:10.1007/s10557-021-07255-2)
Supplement: Supplementary file 1 — Supplementary file1 (DOCX 27 KB) [file 10557_2021_7255_MOESM1_ESM.docx]

**Online Resource 1 Coding definitions used to identify inclusion criteria and outcome variables**

| **Inclusion criteria** | | |
| --- | --- | --- |
| **Myocardial infarction** | **<ICD-10>**  I21 (Acute myocardial infarction)  I22 (Subsequent myocardial infarction)  I23 (Certain current complications following acute myocardial infarction) | |
| **Ischemic stroke** | **<ICD-10>**  I63 (Cerebral infarction)  I64 (Stroke, not specified as hemorrhage or infarction)  I69.3 (Sequelae of cerebral infarction)  **Excluded haemorrhagic stroke**  I60 (Subarachnoid haemorrhage)  I61 (Intracerebral haemorrhage)  I62 (Other nontraumatic intracranial haemorrhage)  **Excluded TIA**  G45 (Transient cerebral ischemic attacks and related syndromes  G46 (Vascular syndromes of brain in cerebrovascular diseases) | |
| **Symptomatic PAD** | Intermittent claudication with ankle-brachial index (ABI) <0.85, or peripheral arterial revascularization procedure, or amputation due to atherosclerotic disease  ***ICD-10 and ABI <0.85***  ***ICD-10 and claim code***  **<ICD-10>**  I70.2 (Atherosclerosis of arteries of extremities)  I73.8 (Other specified peripheral vascular diseases)  I73.9 (Peripheral vascular disease, unspecified)  I74.3 (Embolism and thrombosis of arteries of lower extremities)  **Amputation (claim code)**  N0572 (Amputation of Extremities [Thigh])  N0573 (Amputation of Extremities  [Upper Arm, Forearm, Lower Leg])  N0574 (Amputation of Extremities [Hand, Foot])  N0575 (Amputation of Extremities [Finger, Toe])  **Peripheral arterial revascularization procedure (claim code)**  M6597 (Percutaneous transluminal angioplasty)  M6605 (Percutaneous intravascular installation of metallic stent)  M6613 (Percutaneous intravascular installation of stent-graft)  M6632 (Percutaneous thrombus removal)  M6620 (Percutaneous intravascular atherectomy)  **Open revascularization (claim code)**  O1643, O1644, O1645, O16436,  O0161, O0162, O0163, O0164, O0165, O0166, O0167, O0168, O0169, O0170, O0171 | |
| **Major or minor** | | |
| **Diabetes mellitus** | **<ICD-10>**  E10. (Insulin-dependent, T1DM)  E11. (Non-insulin-dependent, T2DM)  E12. (Malnutrition-related DM)  E13. (Other specified DM)  E14. (Unspecified DM) | |
| **Outcome variable** | |  |
| **All-cause mortality** | **<ICD-10>**  I46.1 (Sudden cardiac death, so described)  I46.9 (Cardiac arrest, unspecified)  R96 (Other sudden death, cause unknown)  R98 (Unattended death)  R99 (Other ill-defined and unspecified causes of mortality)  Documentations of in-hospital mortality |  |
| **Myocardial infarction** | **<ICD-10>**  I21 (Acute myocardial infarction)  I22 (Subsequent myocardial infarction)  I23 (Certain current complications following acute myocardial infarction)  Cardiac enzyme during hospitalization  CAG, PCI or CABG note |  |
| **Stroke and TIA** | **<ICD-10>**  I60 (Subarachnoid haemorrhage)  I61 (Intracerebral haemorrhage)  I62 (Other nontraumatic intracranial haemorrhage)  I63 (Cerebral infarction)  I64 (Stroke, not specified as hemorrhage or infarction)  I69.3 (Sequelae of cerebral infarction)  G45 (Transient cerebral ischemic attacks and related syndromes)  G46 (Vascular syndromes of brain in cerebrovascular diseases)  Reports of CT or MR imaging results |  |
| **Hospitalization for unstable angina** | **<ICD-10>**  I20.0 (Unstable angina)  I24.0 (Coronary thrombosis not resulting in myocardial infarction)  I24.9 (Acute ischemic heart disease, unspecified)  **Excluded chronic stable angina**  I25 (Chronic ischemic heart disease)  CAG, PCI or CABG note  Cardiac enzyme during hospitalization |  |
| **Coronary artery bypass graft surgery** | **Claim code**  OA640, OA641, OA642, OA647, OA648, OA649, O1640, O1641, O1642, O1647, O1648, O1649  CAG, PCI or CABG note |  |
| **Percutaneous coronary intervention** | **Claim code**  **Coronary stenting**  M6561, M6562, M6563, M6564, M6565, M6566, M6567, M6571, M6572  **PTCA**  M6551, M6552, M6553, M6554  CAG, PCI or CABG note |  |

**Online Resource 2 Baseline characteristics for subcohorts of AMC-HR based on intensity of statin treatment and based on index event (MI, IS or sPAD)**

|  | **Lipid-lowering treatment** | | **Index event** | | |
| --- | --- | --- | --- | --- | --- |
|  | Low or moderate  intensity statin | High intensity statin or  any statin with ezetimibe | MI cohort | IS cohort | sPAD cohort |
|  | n=13017 | n=2803 | n=5419 | n=8950 | n=1451 |
| Age, years, mean ± SD | 63.5 ± 10.2 | 62.2 ± 10.0 | 61.3 ± 10.5 | 64.3 ± 9.9 | 63.7 ± 9.4 |
| Men | 8609 (66.1) | 2056 (73.3) | 4121 (76.0) | 5420 (60.6) | 1124 (77.5) |
| Weight, kg, mean ± SD | 64.6 ± 15.1 | 66.0 ± 11.7 | 66.4 ± 18.9 | 64.1 ± 11.6 | 64.4 ± 12.1 |
| BMI, kg/m2, mean ± SD | 24.7 ± 8.3 | 24.9 ± 8.5 | 24.7 ± 7.8 | 24.8 ± 8.2 | 24.3 ± 10.5 |
| SBP, mmHg, mean ± SD | 130.2 ± 21.5 | 127.2 ± 21.5 | 120.5 ± 19.5 | 134.9 ± 20.7 | 130.1 ± 21.9 |
| DBP, mmHg, mean ± SD | 75.1 ± 12.5 | 73.7 ± 12.3 | 71.4 ± 11.6 | 77.0 ± 12.5 | 73.6 ± 12.2 |
| eGFR < 60ml/min/1.73 m2 | 2298 (17.7) | 475 (17.0) | 974 (18.0) | 1388 (15.5) | 411 (28.3) |
| *Lipids, median (IQR)* |  |  |  |  |  |
| LDL-C, mg/dL | 105 (79.0-132.2) | 99.8 (72-130) | 99.4 (75.0-126.4) | 109 (81-136.6) | 95.4 (71-123.1) |
| Total cholesterol, mg/dL | 165 (136-197) | 160 (128-196) | 157 (129-187) | 171 (138-204) | 160 (130-194) |
| HDL-C, mg/dL | 42 (35-51) | 41 (34-49) | 40 (34-48) | 44 (37-53) | 40 (32-49) |
| Triglycerides, mg/dL | 117 (84-163) | 123 (88-176) | 120 (87-167) | 116 (84-164) | 117 (85-170) |
| Lipoprotein(a), nmol/L | 23.3 (12.1-42.8) | 23.1 (12.2-41.8) | 22.4 (11.9-41.0) | 23.9 (12.1-44.2) | 28.2 (13.6-49.4) |
| *Cardiovascular risk factors* |  |  |  |  |  |
| Hypertension | 9780 (75.1) | 2101 (75.0) | 3700 (68.3) | 7045 (78.7) | 1136 (78.3) |
| Diabetes | 5396 (41.5) | 1255 (44.8) | 2131 (39.3) | 3711 (41.5) | 809 (55.8) |
| Current smoking | 2668 (20.5) | 771 (27.5) | 1361 (25.1) | 1658 (18.5) | 420 (28.9) |
| *Cardiovascular disease* |  |  |  |  |  |
| MI | 4232 (32.5) | 1187 (42.3) | 5419 (100.0) | 0 (0.0) | 0 (0.0) |
| IS | 7687 (59.1) | 1263 (45.1) | 0 (0.0) | 8950 (100.0) | 0 (0.0) |
| sPAD | 1098 (8.4) | 353 (12.6) | 0 (0.0) | 0 (0.0) | 1451 (100.0) |
| *Statin use: statin intensity at index event* |  |  |  |  |  |
| Low intensity | 1118 (8.6) | 801 (28.6) | 590 (10.9) | 1037 (11.6) | 292 (20.1) |
| Moderate intensity | 11,899 (91.4) | 1037 (37.0) | 4451 (82.1) | 7429 (83.0) | 1056 (72.8) |
| High intensity | 0 (0.0) | 965 (34.4) | 378 (7.0) | 484 (5.4) | 103 (7.1) |
| *Other medication use* |  |  |  |  |  |
| Any non-statin lipid-lowering treatment |  |  |  |  |  |
| Ezetimibe | 0 (0.0) | 1919 (68.5) | 840 (15.5) | 818 (9.1) | 261 (18.0) |
| Fibrate | 703 (5.4) | 224 (8.0) | 295 (5.4) | 510 (5.7) | 122 (8.4) |
| Niacin | 0 (0.0) | 0 (0.0) | 0 (0.0) | 0 (0.0) | 0 (0.0) |
| Cholestyramine | 11 (0.1) | 6 (0.2) | 6 (0.1) | 7 (0.1) | 4 (0.3) |
| Aspirin or P2Y12 inhibitor | 12,207 (93.8) | 2697 (96.2) | 5390 (99.5) | 8204 (91.7) | 1310 (90.3) |
| Beta blocker | 8246 (63.3) | 2094 (74.7) | 4904 (90.5) | 4445 (49.7) | 991 (68.3) |
| RAAS inhibitor (ACE inhibitor or ARB, aldosterone antagonist, or both) | 9363 (71.9) | 2057 (73.4) | 4304 (79.4) | 6061 (67.7) | 1055 (72.7) |

Data are presented as n (%) unless otherwise indicated

ACE, angiotensin-converting enzyme; ACR, albumin-to-creatinine ratio; AMC-HR, Asan Medical Center Heart Registry; ARB, angiotensin-receptor blocker; BMI, body mass index; CABG, coronary artery bypass graft; DBP, diastolic blood pressure; eGFR, estimated glomerular filtration rate; HDL-C, high-density lipoprotein cholesterol; hsCRP, high-sensitivity C-reactive protein; IQR, interquartile range; IS, ischemic stroke; LDL-C, low-density lipoprotein cholesterol; MI, myocardial infarction; sPAD, symptomatic peripheral artery disease; PCI, percutaneous coronary intervention; RAAS, renin-angiotensin-aldosterone system; SBP, systolic blood pressure; SD, standard deviation
